# Trophic Niche in a Raptor Species: The Relationship between Diet Diversity, Habitat Diversity and Territory Quality

**DOI:** 10.1371/journal.pone.0128855

**Published:** 2015-06-05

**Authors:** Juan Navarro-López, Juan Antonio Fargallo

**Affiliations:** Departamento de Ecología Evolutiva, Museo Nacional de Ciencias Naturales—C.S.I.C., José Gutiérrez Abascal 2, 28006, Madrid, Spain; University of Lleida, SPAIN

## Abstract

Recent research reports that many populations of species showing a wide trophic niche (generalists) are made up of both generalist individuals and individuals with a narrow trophic niche (specialists), suggesting trophic specializations at an individual level. If true, foraging strategies should be associated with individual quality and fitness. Optimal foraging theory predicts that individuals will select the most favourable habitats for feeding. In addition, the “landscape heterogeneity hypothesis” predicts a higher number of species in more diverse landscapes. Thus, it can be predicted that individuals with a wider realized trophic niche should have foraging territories with greater habitat diversity, suggesting that foraging strategies, territory quality and habitat diversity are inter-correlated. This was tested for a population of common kestrels *Falco tinnunculus*. Diet diversity, territory occupancy (as a measure of territory quality) and habitat diversity of territories were measured over an 8-year period. Our results show that: 1) territory quality was quadratically correlated with habitat diversity, with the best territories being the least and most diverse; 2) diet diversity was not correlated with territory quality; and 3) diet diversity was negatively correlated with landscape heterogeneity. Our study suggests that niche generalist foraging strategies are based on an active search for different prey species within or between habitats rather than on the selection of territories with high habitat diversity.

## Introduction

Optimal foraging theory predicts that individuals will select the most favourable habitats for feeding to minimize energy expenditure and maximize fitness [1, 2]. In addition, the classical niche theory predicts a positive correlation between habitat diversity/heterogeneity and diversity of species [3–6]. This is the landscape heterogeneity hypothesis (LHH) [3, 4], and is based on the idea that more heterogeneous landscapes with higher habitat diversity may provide more diverse ways of exploiting the environmental resources (niches) than more homogenous landscapes, consequently allowing exploitation by a greater number of species [5, 7]. Although the relationship between landscape heterogeneity and species diversity has been found in general to be positive, there are cases in which the correlation is not obvious or in which a negative correlation has been observed (see [5] for a review). This discrepancy may be due to factors such as the selected taxonomic group in each study or the size of the effective area for each species [5, 8]. Taking into account both ideas it is plausible to predict that individuals, populations or species with more generalist diets (broader trophic niche) should also exploit more heterogeneous landscapes, as opposed to specialists (narrower trophic niche).

Recent research has indicated that generalist populations are uncommon and that those previously considered generalists may actually be composed of specialist individuals, suggesting the existence of individual ecological specialisation, that is, the degree to which an individual’s diet is restricted relative to their population [9, 10]. Some authors have in fact suggested that ecological specialism is the main driving force leading to speciation, considering generalist strategies as only passing phases in certain evolutionary scenarios [11]. Individuals may specialize on a narrow range of resources, different from those of their conspecific competitors, and thus advantageous by reducing resource-use overlap and competition [9, 12]. Individual specialization is thus expected to be widespread among species occupying higher trophic levels, such as predators, due to a higher intraspecific competition for resources [13–15]. However, generalism can also be adaptive in more unfavourable and/or unpredictable environments, by increasing the capabilities of foraging and the probability for expansion by the colonization of new habitats, hence ensuring persistence [16–19].

In addition to increasing competition, another cost proposed for generalist strategies is the loss of foraging efficiency and, as a result, a reduction of biomass intake compared to more specialist strategies [18, 20, 21]. Under this premise, individuals that are more generalist in a given population should be better able to compensate for these potential costs. A key piece to understanding the evolution of trophic strategies is determining whether the trophic niche is related to individual quality and fitness, which has been little explored in general [17, 22–25]. It is also essential to understand the foraging strategies used by individuals to maximize fitness.

Realized trophic niche (niche that a species occupies when limiting factors, such as interspecific competition, are present) has been recently measured for common kestrels *Falco tinnunculus* in a Mediterranean mountainous area, reporting that individuals showing a broader trophic niche are those of higher quality in the population, as denoted by their higher fecundity (clutch size) and higher offspring survival prospects (better body condition and immune response of the chicks) [17]. In territorial bird species, such as most raptors, breeding performance and foraging behaviour is closely related to territory characteristics [26], since a breeding territory is mainly defined as a defended area for nesting and feeding [27]. In this study, we explore the potential role of territory selection in the trophic niche width of common kestrels during an 8-year period. We analysed diet diversity, territory quality and the diversity of habitats present in territories of common kestrels. Following the LHH we predict that: 1) better territories will be those having higher landscape heterogeneity and 2) since higher quality individuals show a broader trophic niche [17], better individuals placed in better-quality and more heterogeneous territories will consume a higher diversity of prey species.

## Material and Methods

### Study species

The common kestrel (hereafter kestrel) is a territorial diurnal raptor species widely distributed in Eurasia and Africa and common in a broad array of habitats [28]. The variety of environments occupied by kestrels predicts a great variety of foraging habits, with the kestrel considered a rodent specialist in northern populations [29, 30] and preying on a great variety of taxa in more southern populations [17, 31].

### Ethical statement

Our study followed ethical guidelines proposed for the Spanish Royal Decree 1205/2005 on the protection of animals used in experiments and scientific research. Permission to carry out our work was given by Dirección General del Medio Natural de la Junta de Castilla y León. The Spanish Ministry of Science and Innovation (Projects: CGL2007-61395/BOS and CGL2010-15726/BOS) approved the experimental design and financed the study. Common kestrel *Falco tinnunculus* is not considered as endangered species. The research was carried out on private lands with landowner permission.

### Study area and landscape heterogeneity

**Fig 1:**
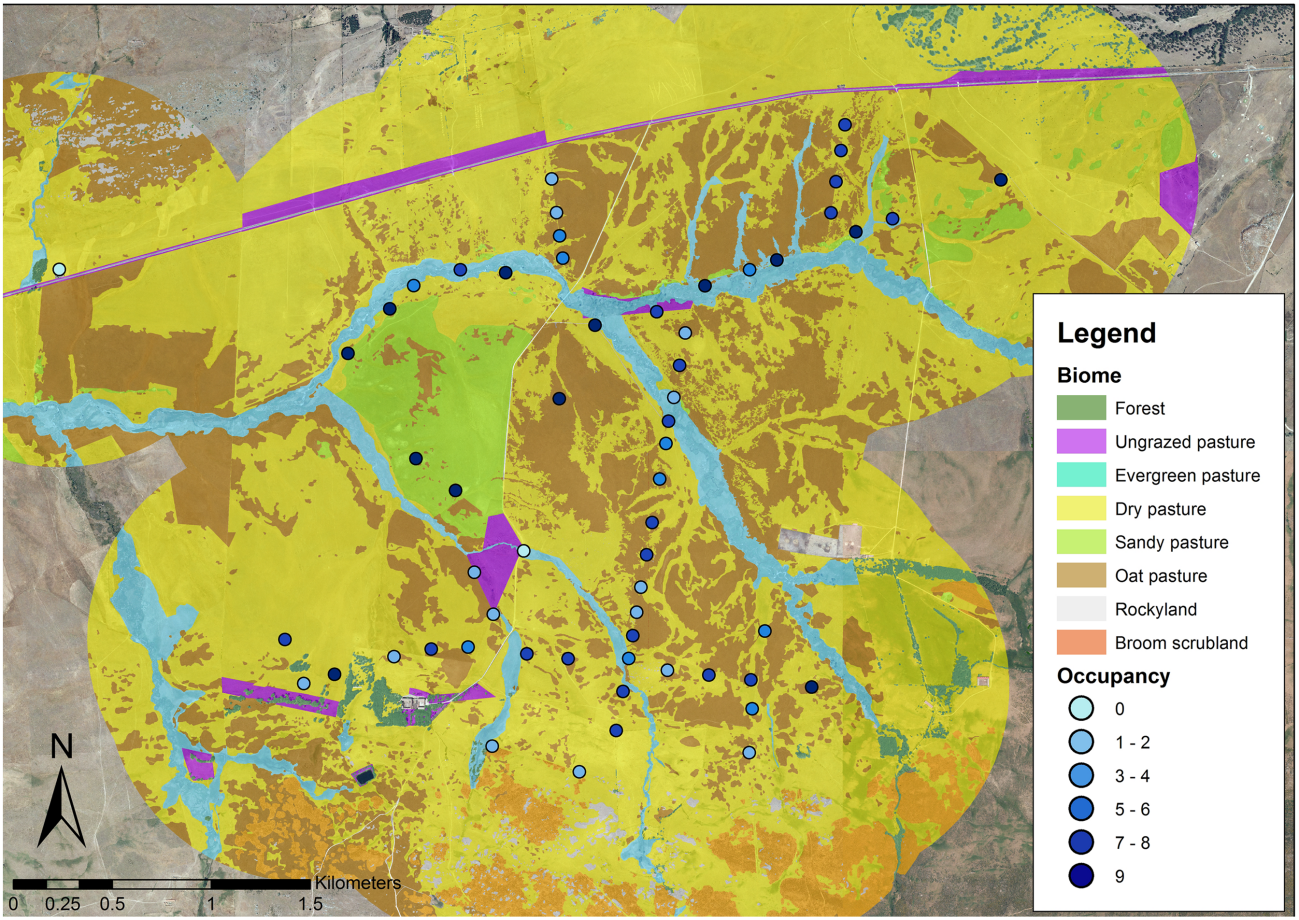
Map of habitats in the study area. Nest boxes are represented by blue dots, blue intensity represents occupancy of the nest.

The study was carried out in the region of Campo Azálvaro, located in central Spain. In this region, about 30–45 breeding pairs nest each year in nest boxes over an area of 23 km^2^ [32]. The area is a flat treeless valley at 1300 m a.s.l., mainly devoted to cattle- raising where pasturelands represent 92% of the habitats, broom scrubland (*Cytisus scoparius*) 5%, small forest fragments (*Populus*, *Fraxinus*, *Salix*, *Pinus* and *Quercus*) 2% and roads, buildings and rocky lands represent around 1% (Fig 1). Vegetation composition of pastures is described in Torre et al. (2007). The pastureland of the study area was characterised into five different habitat types according to vegetation, humidity and ground characteristics: 1) evergreen pastures, defined as pastures with 100% vegetation coverage located around rivers, springs and other water sources that remain green (fresh) throughout the year; 2) dry pasture, defined as pastures with 100% vegetation coverage that become dry in early-middle July; 3) oat pastures, defined as pastures with 100% vegetation coverage that become dry in early-middle July with the presence of golden oat *Stipa gigantea* keeping it green throughout the year; 4) sandy pastures, defined as sparse pastures present in sandy soils where vegetation coverage is not complete; and 5) un-grazed pasture, defined as small areas of pasture with 100% vegetation coverage from which grazing is excluded by means of wire fences.

To determine landscape heterogeneity in kestrel territories the extent of each habitat (pastureland type and the remaining habitats) found in the study area was estimated within an area of a radius of 1000 m around the nest box, 6.3 km^2^ (S1 Table). This area was selected because breeding males hunt in 90% of cases at a distance less than 1 km from the nest in grassland habitats [28]. Orthophotos with maximum resolution were obtained from the “Spanish National Institute of Geography (National Plan of Aerial Orthophotografy 2010)” and analysed in ESRI ArcGIS10Desktop software, Redlands, CA: Environmental Systems Research Institute [33, 34]. Habitat values for each territory (arcsine transformed surface percentage) were combined in a Principal Component Analysis [35, 36] that resulted in 2 main PCs (Table 1). PC1 explained 47% of the variance (eigenvalue = 3.78) and represents a gradient from high values of dried pastures and forest to high values of evergreen and oat pasture. PC2 explained 33% of the variance (eigenvalue = 2.62) and represents a gradient from high values of rocky lands and broom scrubland to high values of un-grazed and sandy pasture.

**Table 1:** Principal components of the composition of habitat around the kestrel nest.

	PC1	PC2
**Forest**	**0.797751**	0.244562
**Rocky land**	0.537136	**0.726177**
**Broom scrubland**	0.579884	**0.707719**
**Evergreen pasture**	**-0.903859**	-0.097197
**Ungrazed pasture**	0.303584	**-0.810392**
**Dry pasture**	**0.852612**	0.242958
**Sandy pasture**	-0.169799	**-0.898918**
**Oat pasture**	**-0.926134**	0.010760
**Eigenvalues**	3.783813	2.621391
**Explained variance**	0.472977	0.327674

Axes selected were all axes with values higher than 1.0 eigenvalues.

Territory landscape heterogeneity (TLH) was defined as the diversity of habitats present in a kestrel territory and was calculated using the Shannon-Wiener index, in the VEGAN package of R version 2.15.2 (2012), by considering habitat richness (number of habitats present) and the expanse (surface percentage) of each habitat type. TLH correlated positively with PC1 and negatively with PC2 (PC1: *r* = 0.33, *F*_2,58_ = 8.63, *P* = 0.005; PC2: *r* = -0.44, *F*_2,58_ = 15.53, *P* < 0.001), which means that territories mainly with higher expanse of forest-dry pasture (PC1) and with sandy-ungrazed pastures (PC2) are the least heterogeneous territories.

### Occupancy and territory quality

Nest boxes were monitored every year from 2005 to 2013 to record kestrel occupation, laying date (the date at which the first egg was laid) and clutch size. Occupancy was defined as the number of years a territory (nest box) was occupied over the 9-year period. Average occupancy was 4.8 ± 2.0 years, ranging from 0 to 9 years (Fig 1). Occupancy was used to estimate territory quality, since good territories are occupied more frequently than bad territories [37]. Controlling for year, occupancy was significantly and negatively correlated with laying date (LMM, *F*_1,222_ = 13.30, *P* < 0.001) and positively correlated with clutch size (LMM, *F*_1,222_ = 6.98, *P* = 0.01), two reliable indicators of individual quality in kestrels [17, 38]. Since occupancy was correlated with clutch size and laying date, we can assume that those territories with a higher occupancy are the territories preferred by individuals of better quality. This is useful to describe territory characteristics (landscape heterogeneity) in relation to territory quality. The number of occupied nest boxes around the nest can affect occupancy. Thus, breeding density was recorded and estimated as the number of breeding pairs within a 1-km radius around the nest box. One of the 62 nest boxes was moved from its place of origin during the study period, so this territory was excluded from the analyses.

### Kestrel diet

From 2006 to 2013, the food provided by parents to their chicks was recorded in 170 nests (16 in 2006, 18 in 2007, 26 in 2008, 25 in 2009, 16 in 2010, 25 in 2011, 21 in 2012 and 23 in 2013). When chicks were 12–14 days old, a digital camera was placed at the nest to record prey delivered by adults when feeding the chicks. The cameras used were: a Cylinder SONY 1/3* Super HAD connected to ARCHOS AV500 100 Gb digital recorders in years 2006–2007; digital camcorders SONY HandyCam 60 Gb in years 2008–2011; and microcameras CCD 1/3 Sharp connected to AXIS Q7401 analogic video encoder in years 2012–2013. The cameras in the first two systems were installed in the posterior wall of the nest box pointing towards the nest box entrance, while in the third system the camera was installed in the lateral wall near the entrance. Both digital recorders and camcorders were powered with 12 amp SLI batteries (24 Ah 24 V) through a voltage converter (12 V). Kestrel nests were recorded continuously for 24 hours or more from sunrise to sunset without researcher interruptions, although some nests were not filmed for the entire period due to technical problems. The daylight period at our study area during June and July is about 15 hours (sunrise at 4:49 hours and sunset 19:49 hours, solar time for 24 June). On average, kestrels began provisioning chicks with food at 7:42 ± 0:58 h solar time (range = 5:30–9:41, n = 133) and stopped at 21:04 ± 0:40 h solar time (range = 19:11–22:09, n = 169). A mean recording time of 16.5 h ± 2.5 h of prey delivery activity was recorded (ranging from 7.7 to 22.4 h, n = 170). Recordings were displayed in the free VLC Media Player software (www.videolan.org) to identify each delivered prey item.

### Diet diversity and individual quality

The diversity of prey delivered by parents was calculated through the Shannon-Wiener index of each nest using the VEGAN package of R [39]. The lowest taxonomic rank was determined in each prey item [40]. Almost all amphibian, reptile, bird and mammal prey items were determined at a species level (99% of cases, Table 2). Among invertebrate prey items, field crickets *Gryllus campestris* and mole crickets *Gryllotalpa gryllotalpa* (*Insecta*, *Orthoptera*) and Mediterranean tarantula *Lycosa tarentula* (*Arachnida*) were easily identifiable in the recordings (Table 2). The rest of the arthropods were identified at the minimum possible taxonomic rank (order and family, Table 2). To calculate Shannon-Wiener index, species level was used for amphibians, reptiles, mammals, birds, spiders, crickets and mole crickets, family for grasshopper, bush crickets and mantises and order for beetles, butterfly and moth larvae. Diversity is expected to vary with the sampling effort [41]. In our case, diversity of diet was not associated with the filming time for the range we worked with (LMM, *R* ^*2*^ *c* = 0.26, *F*_1,101_ = 1.34, *P* = 0.25). As in the previous four-year study period [17], diet diversity was positively correlated with clutch size for the longer eight-year period in this study (LMM, *R* ^*2*.^ *c* = 0.30, F_1,108_ = 8.58, *P*< 0.004; see below for statistical details). Similarly indicating an association between kestrel diet diversity and individual quality.

**Table 2:** Total numbers and percentage of prey items delivered by common kestrel *Falco tinnunculus* parents to the nest over an eight-year study period (2006–2013).

	N (%)
**Mammals**	
*Apodemus sylvatica*	8 (0.09)
*Crocidura russula*	63 (0.68)
*Microtus arvalis*	779 (8.45)
*Talpa occidentalis*	1 (0.01)
**Birds**	
*Alauda arvensis*	25 (0.27)
*Anthus campestris*	2 (0.02)
*Carduelis cannabina*	2 (0.02)
*Lanius senator*	1 (0.01)
*Motacilla flava*	2 (0.02)
*Passer domesticus*	5 (0.05)
*Petronia petronia*	3 (0.03)
*Sturnus unicolor*	26 (0.28)
Passerines (unidentified)	24 (0.26)
**Reptiles**	
*Chalcides striatus*	248 (2.69)
*Timon lepidus*	311 (3.37)
*Lacerta schreiberi*	64 (0.69)
*Podarcis hispanica*	57 (0.62)
*Psammodromus hispanicus*	1461 (15.85)
Large Lizard	6 (0.07)
Small lizard	5 (0.05)
**Amphibians**	
*Triturus marmoratus*	3 (0.03)
*Pelobates cultripes*	7 (0.08)
*Pelophylax perezi*	27 (0.29)
**Arthropods**	
*Lycosa tarentula*	41 (0.44)
*Gryllus campestris*	1569 (17.02)
*Acrididae*	229 (2.48)
*Tettigoniidae*	246 (2.67)
*Gryllotalpa gryllotalpa*	3309 (35.9)
*Orthoptera (unidentified)*	5 (0.05)
*Mantodea*	3 (0.03)
*Neuroptera*	1 (0.01)
*Lepidoptera*	1 (0.01)
*Coleoptera*	482 (5.23)
*Insecta (unidentified)*	107 (1.16)
*Insecta* (*larvae*)	70 (0.76)
Unidentified prey item	24 (0.26)
**Total prey items**	**9217**

The minimum identified taxon level is shown.

### Statistical procedures

Statistical analyses were performed using R, version 2.15.2 (CRAN 2012). The relationship between occupancy and territory habitat characteristics (diversity and PCs) was analysed using general lineal models (LM). In a first step the relationship between occupancy (dependent variable) and territory landscape heterogeneity, TLH, (independent variable) was analysed in order to explore how territory quality varies with landscape heterogeneity (first prediction). The squared term of habitat diversity was included as an independent variable to test for a possible curvilinear relationship. In a second LM, occupancy was correlated with principal components of territory habitat (PC1 and PC2), breeding density and habitat diversity to know other environmental characteristics associated with territory quality.

Diet diversity was analysed using general linear mixed models (LMM). In a first LMM, the relationship between diet diversity and clutch size was explored. Since clutch size and laying date were closely correlated (LMM *R* ^*2*.^ *c* = 0.17, *F*_1,101_ = 24.78, *P*< 0.001) the effect of laying date on clutch size was removed by including the residuals of clutch size on laying date as an independent variable. Year and nest were included as random factors. Once this association was verified, a second LMM was done to test the second prediction: a more diverse diet in good-quality individuals is obtained from a more heterogeneous territory. For this purpose diet diversity was included in the model as the dependent variable, and TLH, habitat PC1 and PC2 as covariates. Since prey abundance and availability changes as the season progresses, laying date was also included in the model as a covariate. Year and nest were included as random factors.

LMs and LMMs were performed with the *lme4* R package (CRAN 2013)[42] and statistics were obtained with the *lmerTest* R package (CRAN 2013) [43]. Residuals obtained from all LM and LMMs showed normal distributions (Shapiro-Wilk, all *P* > 0.05). R^2^ conditional [44] was calculated using the *MuMln* R package (CRAN 2014) [45]. We constructed sets of models with possible combinations of independent variables. Akaike’s information criterion corrected for small sample size (*AICc*) was used for model selection. The best model was the one with the lowest *AICc* value with a difference > 2 from the second best model. *ΔAICc* and *AICc* weights were also calculated.

## Results

### Occupancy and landscape heterogeneity

**Fig 2:**
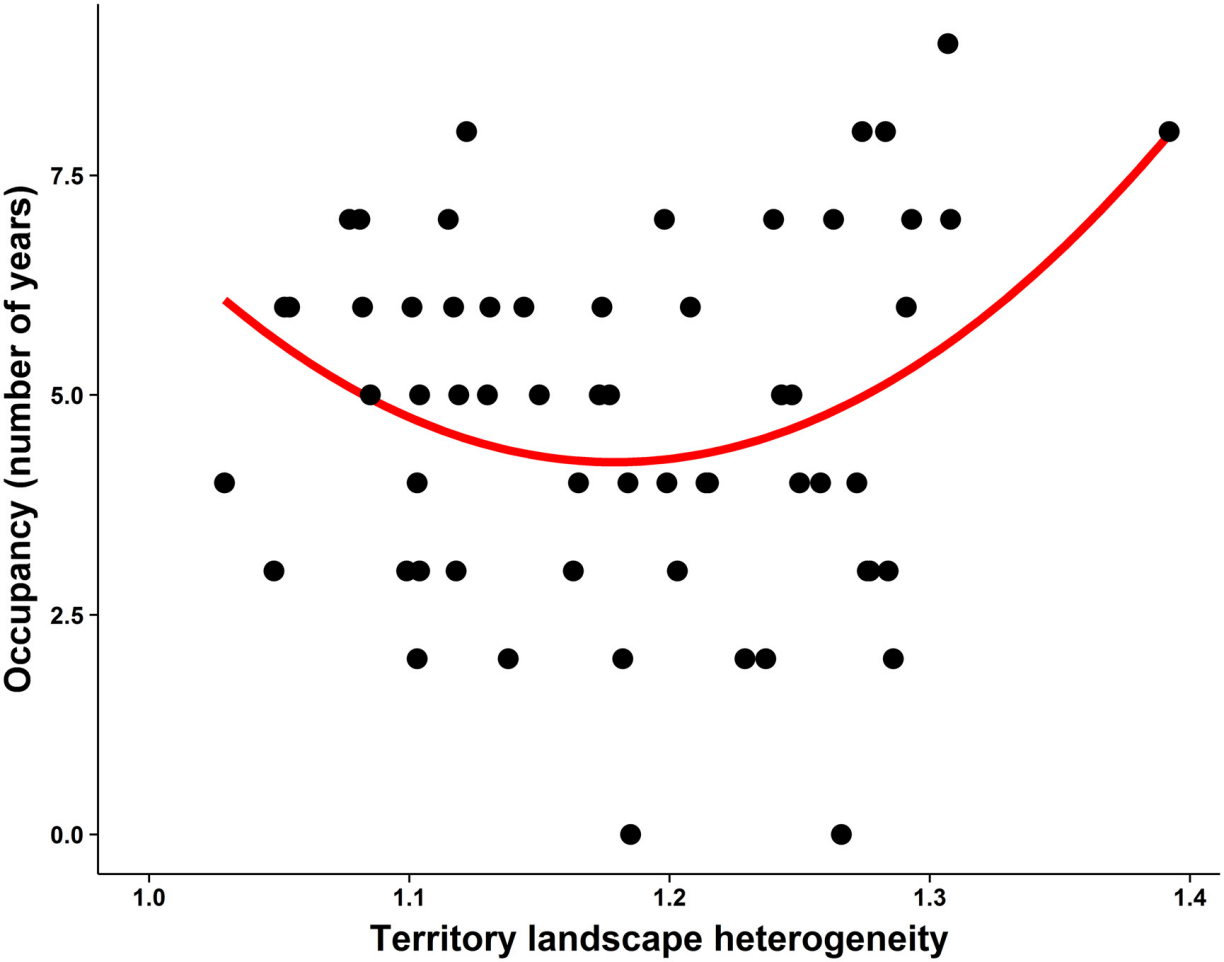
Quadratic relationship between nest box occupancy and territory landscape heterogeneity (Shannon-Wiener index of habitats) of common kestrels.

Dry pasture was the most widespread habitat in the study area (58.13%,) followed by oat pasture (22.12%, Fig 1 and S1 Table). These two habitats were also the most commonly found in the 1km-radius areas where nest boxes were installed (53.7% and 27.4% respectively; S1 Table). On average, habitat diversity in kestrel territories was 1.18 ± 0.08, ranging from 1.03 to 1.39. The LM exploring the relationship between occupancy and TLH showed a curvilinear correlation (TLH, *F*_1,58_ = 5.44, *P* = 0.023, TLH^2^, *F*_1,58_ = 5.54, *P* = 0.02; Fig 2). The highest occupancy was observed in the most and least diverse territories. When including in the model habitat PC1, habitat PC2 and breeding density as other potential explanatory variables, the best model obtained for this set of variables was the one containing the terms PC2, TLH and TLH^2^ (Table 3). Occupancy was significantly and negatively correlated with PC2 (Table 3), indicating that more frequently occupied territories were those with larger expanses of ungrazed and sandy pastures and shorter expanses of rocky lands and scrubland. In the selected model the effects of TLH and TLH^2^ were statistically reduced (Table 3). Occupancy was not significantly correlated with either breeding density or habitat PC1 (both *P* > 0.11).

**Table 3:** Best general linear model (LM) of the occupancy of nests, as a measure of territory quality.

Effect	Estimate	SE	*F*	*P*	95% CI
TLH	-138.8575	80.52	2.97	0.090	(-300.096, 22.378)
TLH^2^	57.4116	33.97	2.86	0.097	(-10.609, 125.432)
Habitat PC 2	-0.0085	0.01	8.41	0.005	(-0.014, -0.003)

Degrees of freedom = 56, *n* = 61, R^*2*^ conditional = 0.20, estimates, standard errors (SE), *F* and *P* values are shown. (*AICc* for the initial model = 252.8, *AICc* for the second best model = 245.3, *AICc* for the best model = 242.9, *ΔAICc* = 2.4).

### Diet, occupancy and landscape heterogeneity

As shown in Table 2, arthropods, mainly Orthoptera, were the most frequently consumed prey group by kestrels in the population, followed by mammals, reptiles, birds and amphibians. Within species, the mole cricket, field cricket, Spanish psammodromus *Psammodromus hispanicus*, common vole *Microtus arvalis*, ocellated lizard *Timon lepida* and Western three-toed skink *Chalcides striatus* were the six most preyed upon species (83% of prey items). Prey provisioning rate was 3.3 ± 2.1 prey items / h, ranging from 0.3 to 10.0.

**Fig 3:**
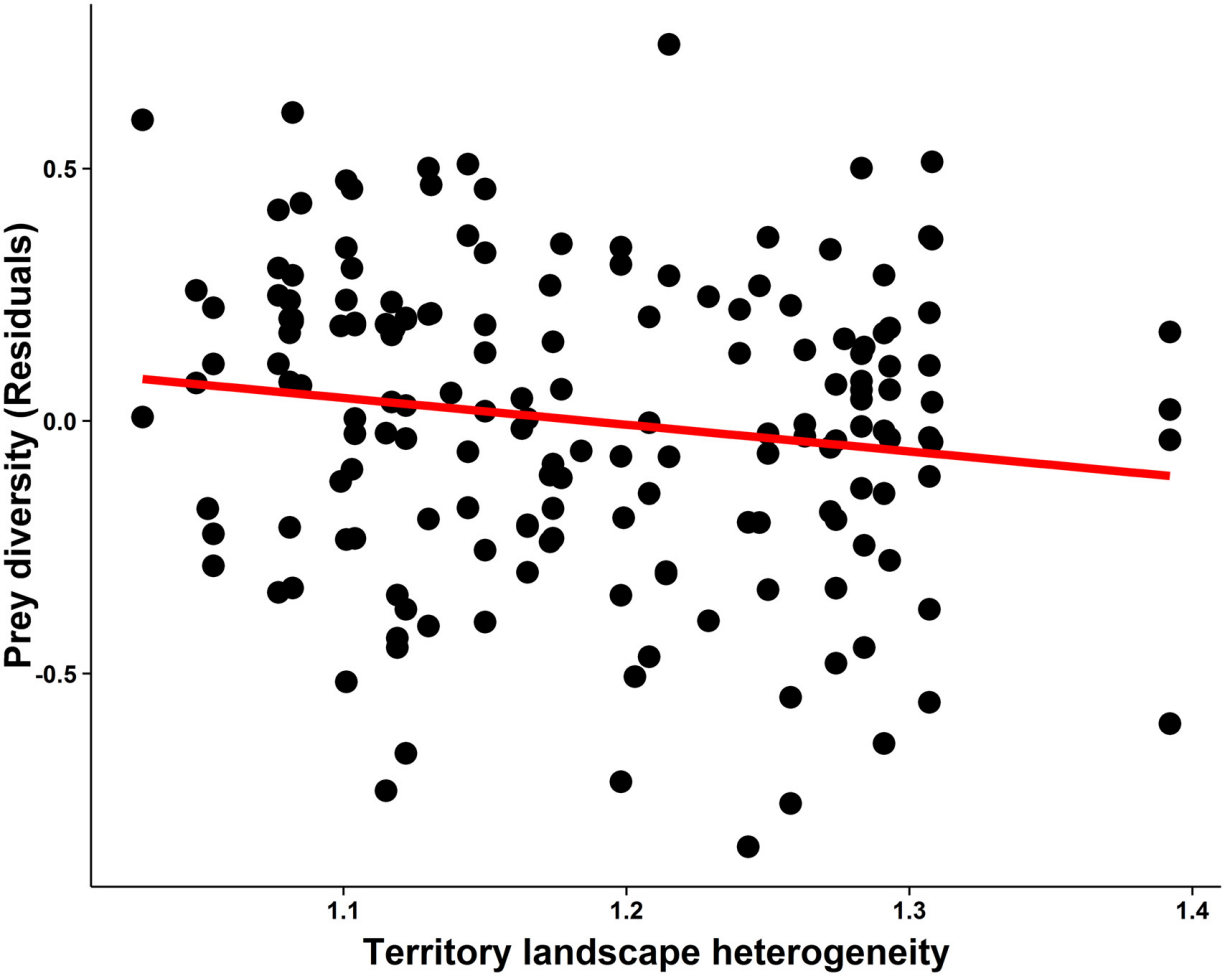
Relationship between diet diversity (residuals) of common kestrels and territory landscape heterogeneity. Residuals were extracted by excluding territory landscape heterogeneity from the model.

The mean kestrel diet diversity for the 8-year period was 1.32 ± 0.38, ranging from 0.26 to 2.24 Shannon-Wiener index. The model selection procedure for diet diversity yielded two best models with similar *AICc* (Table 4). Both models showed that controlling for the effect of laying date, diet diversity was negatively correlated with TLH. The diversity of prey consumed was higher in territories with a lower diversity of habitats (Fig 3). Occupancy and habitat PCs did not show statistical significant effects on diet diversity (Table 4).

**Table 4:** Best general linear mixed models (LMMs) of diet diversity in common kestrels (Shannon-Wiener index).

Effect	Estimate	SE	*F*	*P*	95% CI
**1** ^**st**^ **best model**					
Laying date	0.0142	0.00	35.25	< 0.001	(0.009, 0.019)
TLH	-0.7600	0.34	5.13	0.025	(-1.425, -0.095)
Habitat PC 2	-0.0402	0.03	1.79	0.183	(-0.099, 0.019)
**2** ^**nd**^ **best model**					
Laying date	0.0146	0.01	37.10	<0.001	(0.010, 0.019)
TLH	-0.8208	0.34	5.91	0.017	(-1.490, -0.151)
Occupancy	0.0185	0.02	1.49	0.224	(-0.012, 0.049)
Habitat PC 2	-0.0342	0.03	1.28	0.260	(-0.094, 0.026)

Year and nest were included as random factors. Degrees of freedom = 108, *n* = 170, *R* ^*2*^ conditional = 0.31, estimates, standard errors (SE), *F* and *P* values are shown. (*AICc* for the initial model = 107.5, *AICc* for the second best model = 105.3, *AICc* for the first best model = 104.6, *ΔAICc* = 0.7).

## Discussion

Landscape heterogeneity promotes an increase in species diversity (LHH), and thus a higher diversity of habitats in kestrel territories should provide them with a greater diversity of prey in the diet. Diet diversity in this kestrel population has been observed to be positively correlated with indicators of individual quality, such as body condition and immunity of offspring and clutch size ([17], this study). Accordingly, if territory reflects individual quality, it is feasible to expect a positive correlation between habitat diversity and territory quality. Identifying territory quality of birds through occupancy has been successful for many species [37], although this relationship has not been as obvious for others [46, 47]. In kestrels, those individuals showing earlier laying dates and larger clutch sizes are those of better quality [17, 38]. In addition, first occupied territories are those where individuals start breeding earlier and lay larger clutches. Furthermore, these territories were more frequently occupied than others (this study). Thus, without knowing particular territory characteristics providing quality, it is feasible to conclude that for some reason preferred territories are of better quality.

When the relationship between territory quality and territory landscape heterogeneity was analysed, the model showed that contrary to our linear prediction occupancy varied quadratically with territory landscape heterogeneity, with the most frequently occupied territories having the highest and lowest landscape heterogeneity. Since a higher heterogeneity of habitats in nest surroundings does not provide a higher diversity of food for kestrels (see below), selecting more heterogeneous territories may be advantageous for kestrels in regions with drastic spatio-temporal changes in food availability, such as the Mediterranean region [32, 48, 49]. This is due to the fact that more heterogeneous landscapes provide a wider range of alternative prey species that can benefit kestrels when changing environmental circumstances affect the abundance of the preferred prey [48]. At the other extreme, high occupancy rate was also observed in the least diverse territories. This may be due to a preference of kestrels for ungrazed-sandy pastures and/or forest-dry pastures, as deduced from the correlation found between TLH and PCs. In fact, when the structure of the landscape (PCs) is included in the model, the quadratic effect of territory landscape heterogeneity is reduced and the model showed that kestrels selected territories with a higher expanse of ungrazed and sandy pastures avoiding areas with rocks and scrubs, as concluded by the correlation found between occupancy and PC2. We must also note that PCs describe the spatial structure of landscape where nest boxes were installed in our study area. Within this structure, those areas including sandy pastures are also the most distant areas from scrublands and rocky lands, which may also explain the PC2 gradient and the habitat selection of kestrels in our population.

Also contrary to our prediction, the diversity of prey consumed by kestrels was not positively, but negatively correlated with territory landscape heterogeneity. Several conclusions can be drawn from this result. Kestrels do not increase diet diversity by selecting more diverse landscape, but by actively searching for different prey species in less diverse territories. The second conclusion is that forest-dry pastures and sandy-ungrazed pastures (the main habitats represented in less diverse territories) provide the higher diversity of prey species for kestrels. Also, high landscape heterogeneity can result in patches not suitable for hunting. It should be noted that our study approach was based on a mechanistic view of individual specialisation with regard to habitat exploitation so that for each particular habitat, each particular individual is expected to search for one or several particular prey species. This approach, which allowed us to predict a more diverse diet in a more diverse landscape derived from the LHH, supposes a first step to investigate the relationship between trophic niche and habitat use. The same habitat can be occupied by different prey species and prey availability (difficulty of capture) can be different for different species occupying the same habitat type and also for the same species occupying different habitat types [50–52]. In addition, the inter-annual fluctuation in the abundance of prey species also changes hunting behaviour of kestrels [17, 32, 53] and the interaction between habitat and prey availability. All these interactions should be investigated in future studies to account for the relationship found in this study between habitat heterogeneity of territories and kestrel diet.

In our kestrel population individuals adopting a more generalist strategy seem to be able to produce offspring with better body and immunological conditions, and hence with a higher fitness potential, as it was found for other bird species [24, 25]. Broadening the trophic niche may be an adaptive strategy in environments where the abundance and availability of food resources fluctuate with time, such as in Mediterranean regions. An interesting aspect to understanding foraging strategies is the nutritional and biomass value for the diet, as diets based on large and less mobile prey species might be more energetic in terms of biomass and hunting effort than a diverse diet based on small prey species. In a previous study carried out in our population [17] a positive correlation between diet diversity and prey biomass was found, so that individuals consuming a greater variety of prey species also preyed on the larger and heavier prey species. Knowing the nutritional components of the different prey species will be key to understanding costs and benefits associated with foraging strategies.

Our results also showed that the diversity of the consumed prey species was not predicted by territory quality, as no correlation was found between diet diversity and occupancy. This suggests that other characteristics besides food availability are important in territory selection. Nest predation is a major selective force in the reproductive strategies of birds, since it is considered a primary source of nesting mortality [54] and influences the choice of nest sites in small raptors including kestrels [55]. The design and location of the nest boxes in our study area were planned to minimize the risk of predation. Predation events by mammals in the first years [56] had been prevented since 1998 (some original nest box placements were avoided), and sporadic predation by eagle owls *Bubo bubo* occurred in some years during the study period. In our population, kestrels avoided nest boxes close to bush areas or forest fragments (pers. obs.) where the visibility of potential predators is low and the nest is more vulnerable to predation [57]. This selective pressure can also explain the correlation found between occupancy and PC2. In conclusion, our study shows that territory quality does not show a linear relationship with territory landscape heterogeneity, but a curvilinear correlation in which the most and the least diverse territories are occupied at higher rates. In addition, kestrels preferred territories with greater expanses of sandy and ungrazed pastures. Our study revealed that diet diversity in a bird species was associated with landscape characteristics. Contrary to predicted, birds may show higher diet diversity in landscapes with a lower diversity of habitats. In this mountainous Mediterranean pastureland two main habitat types, those combining sandy and ungrazed pastures and also those dry pastures close to forests islets provided the highest diversity in the kestrel diet indicating that kestrels actively search for a diversity of prey species as a foraging strategy. Furthermore, our results suggest that other territory characteristics in addition to food availability, such as possibly predation risk, play an important role in territory and nest-site selection for birds. Finally, this study provides further support to the idea that the frequency of nest occupation can be a good measurement of territory quality in birds [37] as concluded from its correlation with clutch size and laying date in our kestrel population.

## Supporting Information

S1 Table Habitats found in the study area and common kestrel territories. Percentages in the total study area, their average around the used kestrel territories and their average around the high quality territories (occupied six or more times).
(DOC)
