# Supplementary material for: Trophic Niche in a Raptor Species: The Relationship between Diet Diversity, Habitat Diversity and Territory Quality
Source: PLoS One. 2015 Jun 5;10(6):e0128855. doi: 10.1371/journal.pone.0128855 (PMC4457527; doi:10.1371/journal.pone.0128855)
Supplement: S1 Table — Percentages in the total study area, their average around the used kestrel territories and their average around the high quality territories (occupied six or more times). (DOC) [file pone.0128855.s001.doc]

**Supporting Information Legends**

**S1 Table**. Habitats found in the study area and common kestrel territories. Percentages in the whole study area, their average around the used kestrel territories and their average around the high quality territories (occupied six or more years).

| **Habitat** | **Study area** | **Kestrel territories** |
| --- | --- | --- |
| **Dry pasture** | 58.13 | 53.53 |
| **Oat pasture** | 22.12 | 27.78 |
| **Evergreen pasture** | 6.25 | 7.64 |
| **Sandy pasture** | 3.85 | 5.39 |
| **Broom scrubland** | 4.66 | 2.03 |
| **Ungrazed pasture** | 1.94 | 2.01 |
| **Forest** | 1.77 | 1.1 |
| **Rockyland** | 1.04 | 0.55 |
